# Supplementary material for: Two independent modes of kidney stone suppression achieved by AIM/CD5L and KIM-1
Source: Commun Biol. 2022 Aug 3;5:783. doi: 10.1038/s42003-022-03750-w (PMC9349198; doi:10.1038/s42003-022-03750-w)
Supplement: Supplementary file 2 — Supplementary Information [file 42003_2022_3750_MOESM2_ESM.pdf]

Supplementary information for

Two independent modes of kidney stone suppression achieved by AIM/CD5L and KIM-1

Kyohei Matsuura, Natsumi Maehara, Ayaka Eguchi, Aika Hirota, Keisuke Yasuda, Kaori Taniguchi, Akemi Nishijima, Nobuyuki Matsuhashi, Yoshiyuki Shiga, Rumi Ishii, Yasuhiro Iguchi, Kazunari Tanabe, Satoko Arai & Toru Miyazaki

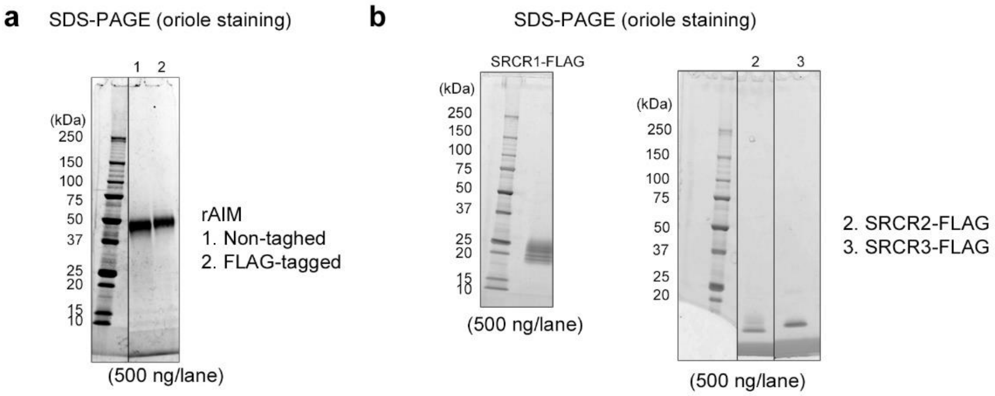

Supplementary Fig.1. (a) Purified rAIM (non-tagged and FLAG-tagged) were separated by SDS-PAGE in reducing condition and stained with Oriole fluorescent gel stain (Bio-Rad). **(b)** Same analysis described in (a) was performed for FLAG-tagged recombinant SRCR proteins.

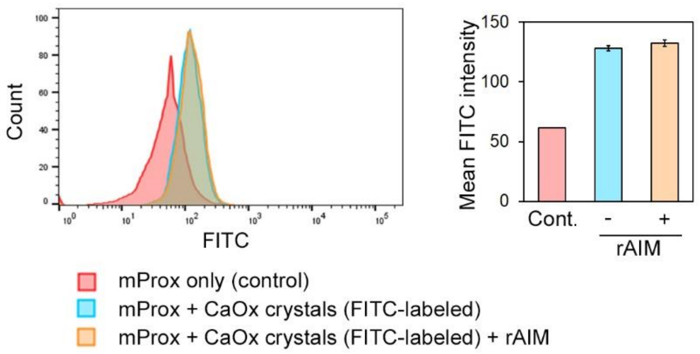

Supplementary Fig. 2. The mProx24 cells were challenged with FITC-labelled CaOx crystals in culture in the presence or absence rAIM (100 μg/mL) at 37°C for 1 h. Crystals were washed and thereafter, the attachment/incorporation of crystals to the cells was analyzed using flowcytometer.

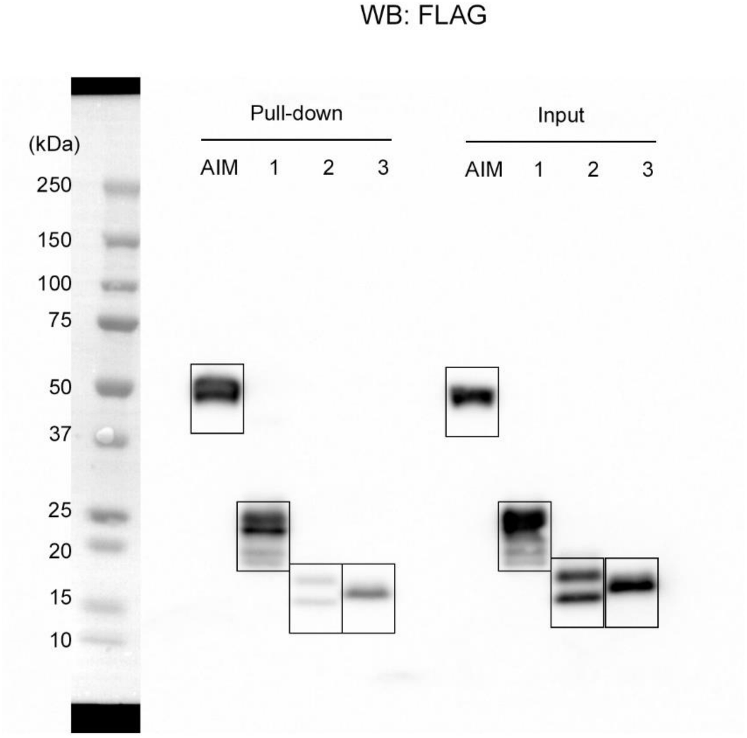

Supplementary Fig. 3. Full-length images of immunoblots for AIM presented in Fig. 2c. The AIM signals are surrounded by squares.

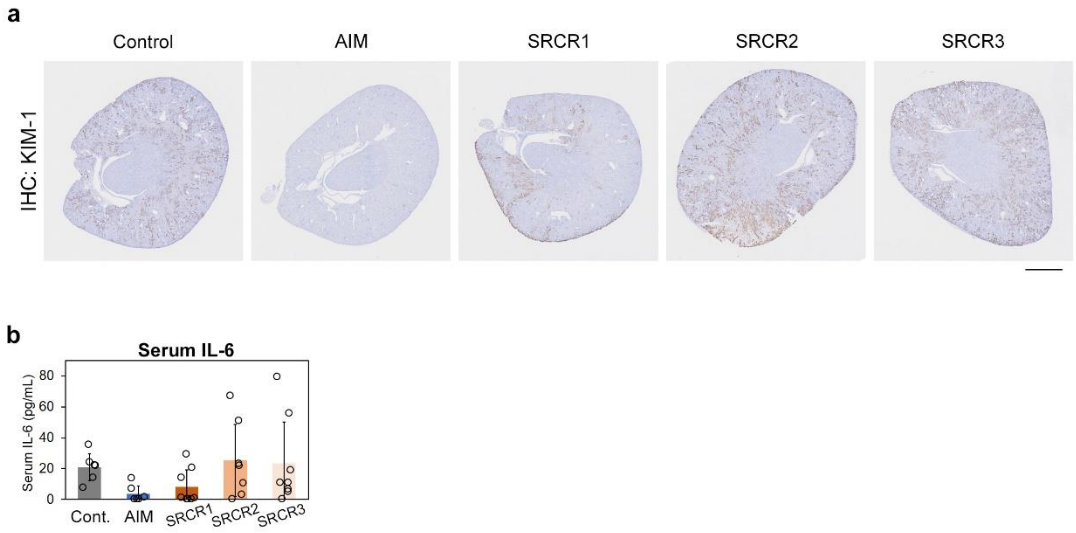

Supplementary Fig. 4. (a) Representative photos for IHC analysis of kidney specimens for KIM-1 in mice at day 6 of glyoxylate loading with injection of FLAG-tagged rAIM (400 µg) or SRCR domain (120 µg for each) on days 1, 3 and 5. Scale bar: 1 mm. **(b)** ELISA analysis of sera from mice above for IL-6. n = 6~8 each. These protein data are the complementary results for the mRNA data presented in Fig. 2f.

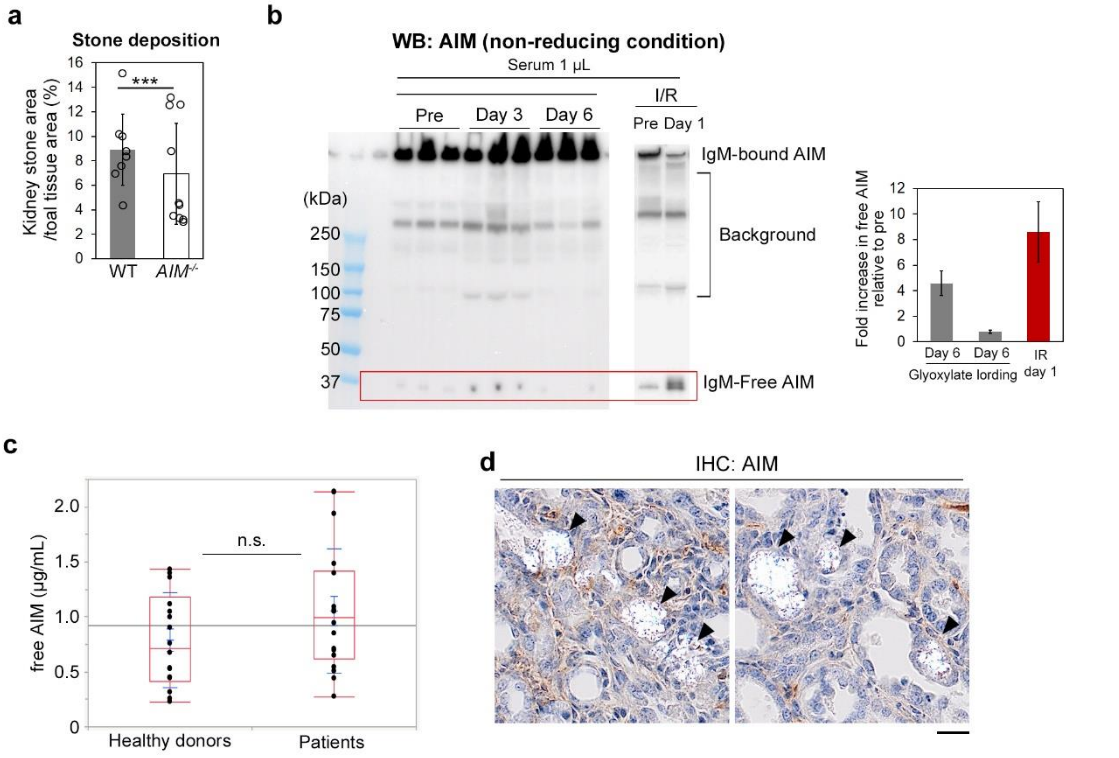

Supplementary Fig. 5. (a) The amount of kidney stones at day 6 of glyoxylate loading in wild-type (n = 7) and $AIM^{-/-}$ mice (n = 8). **(b)** The sera of glyoxylate-loaded mice (n = 3) were Immunoblotted for AIM in non-reducing condition at day 0 (pre) day 3, and day 6. As a reference, sera from mice at day 1 after a challenge with ischemia/reperfusion to induce AKI (indicated as IR) were also analyzed for AIM. Three mice were challenged with IR, and a representative blot is presented (right). IgM-free AIM is surrounded by red square. Fold increases (average ± s . d.) of IgM-free AIM in three glyoxylate-loaded mice (day 3 and day 6) and three IR-challenged mice (day 1) are shown by graphs. **(c)** Sera from human individuals with or without kidney stones were analyzed for IgM-free AIM by ELISA as described previously[1]. n = 18 each. No statistical significance was obtained after analyzed by Welch's t-test. **(d)** Immunohistochemistry for AIM in kidney specimens from mice at day 6 of glyoxylate loading. No AIM staining (brown) was observed at the stone area. Black arrows: stones.

Some interstitial macrophages that express AIM were stained for AIM. Scale bar: 20 μm.

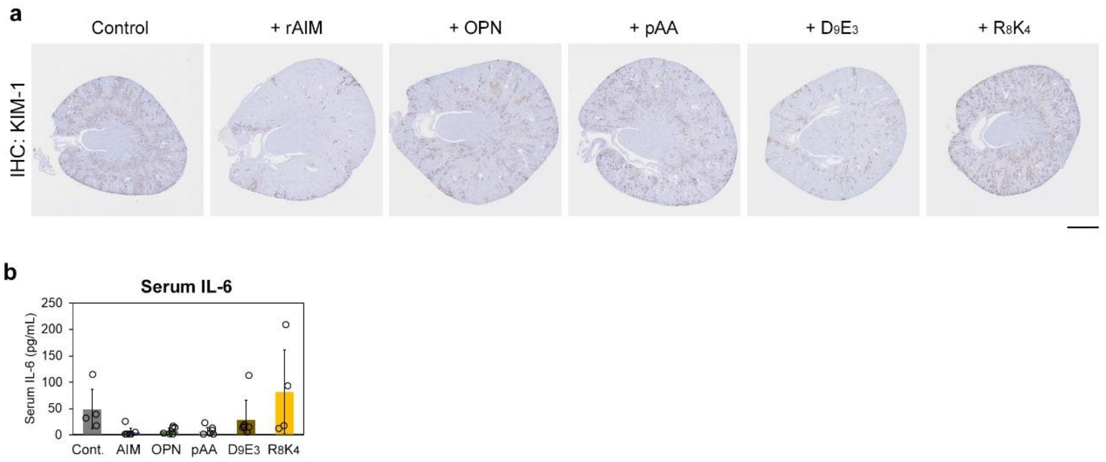

Supplementary Fig. 6. (a) Representative photos for IHC analysis of kidney specimens for KIM-1 in mice at day 6 of glyoxylate loading with injection of the indicated substance at identical molar levels (rAIM; 400 µg, rOPN; 500 µg, $pAA_{5.1}$; 60 µg, D_9E_3 and R_8K_4; 10 µg each) on days 1, 3 and 5. Scale bar: 1 mm. **(b)** ELISA analysis of sera from mice above for IL-6. n = 4~6 each. These protein data are the complementary results for the mRNA data presented in Fig. 3d.

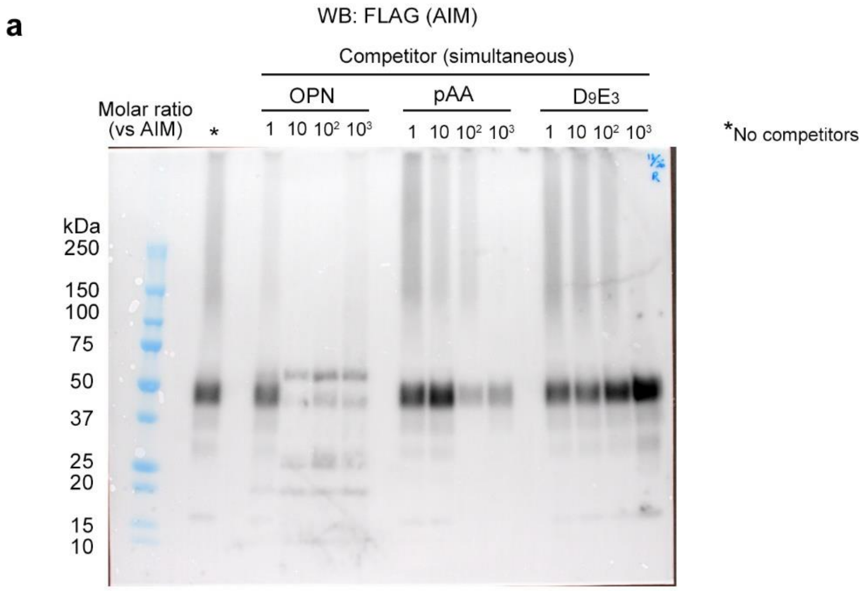

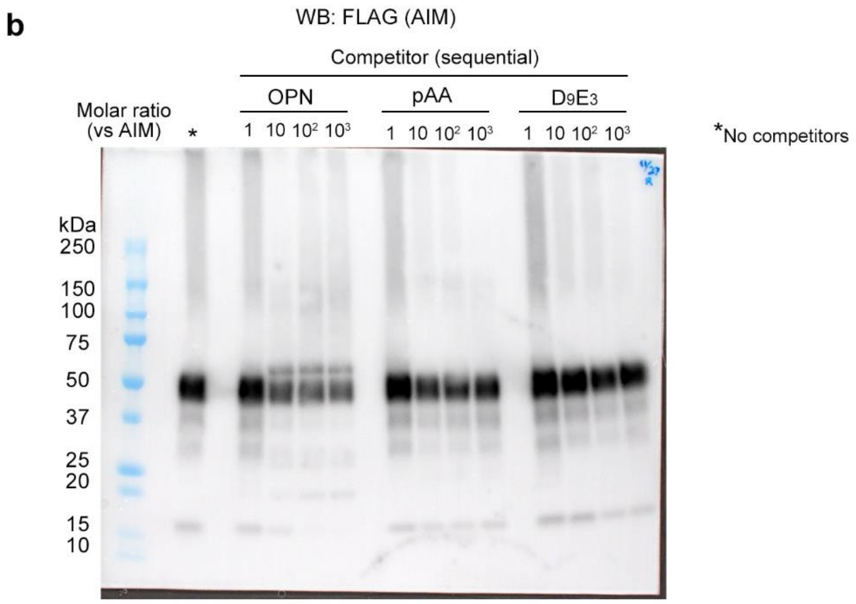

Supplementary Fig. 7. Full-length images of immunoblots for Fig. 4.

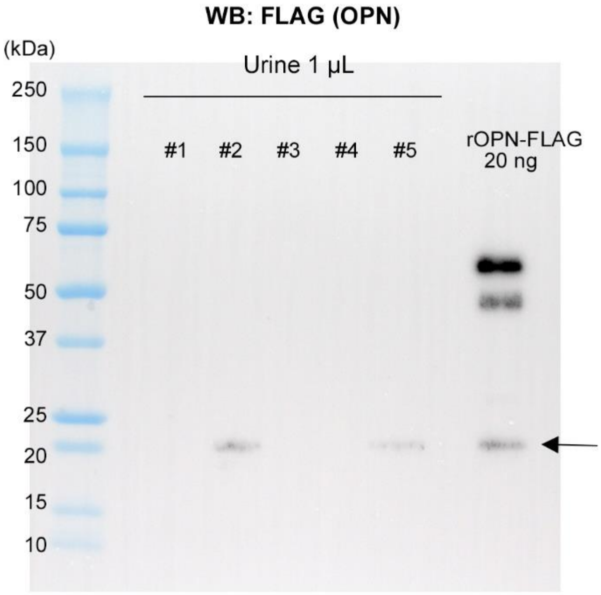

Supplementary Fig. 8. The urine from five glyolxylate-loaded mice (at day 5) i.v. injected with rOPN that was tagged with a FLAG sequence at the C-terminal was pooled for 4 hrs and was analyzed for OPN by Immunoblotting using an anti-FLAG antibody. n = 5. Only a signal of small size corresponding to the truncated rOPN C-terminal fragment[2] appeared (indicated by arrow). Note that the molecular wight of full-size OPN is 45–66 kDa, depending on glycosylation levels.

Supplementary Table 1.

Sequences of the oligonucleotides used for Quantitative PCR assay.

Name	Sequence (5'→3')
f-GAPDH	AGAACATCATCCCTGCATTC
r-GAPDH	CACATTGGGGGTAGGAACAC
f-KIM-1	TCCACACATGTACCAACATCAA
r-KIM-1	GTCACAGTGCCATTCCAGTC
f-NGAL	CCATCTATGAGCTACAAGAGAACAAT
r-NGAL	TCTGATCCAGTAGCGACAGC
f-IL1B	TGTAATGAAAGACGGCACACC
r-IL1B	TCTTCTTTGGGTATTGCTTGG
f-IL6	ATGGATGCTACCAAACTGGAT
r-IL6	TGAAGGACTCTGGCTTTGTCT
f-TNFA	ACGGCATGGATCTCAAAGAC
r-TNFA	AGATAGCAAATCGGCTGACG
f-MCP-1	TGATCCCAATGAGTAGGCTGGAG
r-MCP-1	ATGTCTGGACCCATTCCTTCTTG
f-CD11B	ATGGACGCTGATGGCAATACC
r-CD11B	TCCCCATTCACGTCTCCCA
f-F4/80	CCTGGACGAATCCTGTGAAG
r-F4/80	GGTGGGACCACAGAGAGTTG

Supplementary References

1. Koyama, N.et al. Activation of apoptosis inhibitor of macrophage is a sensitive diagnostic marker for NASH-associated hepatocellular carcinoma. J. Gastroenterol. **53,** 770-779 (2018).

2. Kubota, T., et al. Multiple forms of SppI (secreted phosphoprotein, osteopontin) synthesized by normal and transformed rat bone cell populations: regulation by TGF-beta. *Biochem. Biophys. Res. Commun.* **162,** 1453-1459 (1989).
