# Supplementary material for: Two independent modes of kidney stone suppression achieved by AIM/CD5L and KIM-1
Source: Commun Biol. 2022 Aug 3;5:783. doi: 10.1038/s42003-022-03750-w (PMC9349198; doi:10.1038/s42003-022-03750-w)
Supplement: Supplementary file 3 — Reporting Summary [file 42003_2022_3750_MOESM3_ESM.pdf]

nature portfolio

Corresponding author(s): Toru Miyazaki

Last updated by author(s): 2022/07/20

Reporting Summary

Nature Portfolio wishes to improve the reproducibility of the work that we publish. This form provides structure for consistency and transparency in reporting. For further information on Nature Portfolio policies, see our Editorial Policies and the Editorial Policy Checklist.

Statistics

For all statistical analyses, confirm that the following items are present in the figure legend, table legend, main text, or Methods section.

n/a	Confirmed	
☐	☒	The exact sample size (*n*) for each experimental group/condition, given as a discrete number and unit of measurement
☐	☒	A statement on whether measurements were taken from distinct samples or whether the same sample was measured repeatedly
☐	☒	The statistical test(s) used AND whether they are one- or two-sided<br>*Only common tests should be described solely by name; describe more complex techniques in the Methods section.*
☐	☒	A description of all covariates tested
☐	☒	A description of any assumptions or corrections, such as tests of normality and adjustment for multiple comparisons
☐	☒	A full description of the statistical parameters including central tendency (e.g. means) or other basic estimates (e.g. regression coefficient) AND variation (e.g. standard deviation) or associated estimates of uncertainty (e.g. confidence intervals)
☐	☒	For null hypothesis testing, the test statistic (e.g. *F*, *t*, *r*) with confidence intervals, effect sizes, degrees of freedom and *P* value noted<br>*Give P values as exact values whenever suitable.*
☒	☐	For Bayesian analysis, information on the choice of priors and Markov chain Monte Carlo settings
☒	☐	For hierarchical and complex designs, identification of the appropriate level for tests and full reporting of outcomes
☒	☐	Estimates of effect sizes (e.g. Cohen's *d*, Pearson's *r*), indicating how they were calculated

Our web collection on statistics for biologists contains articles on many of the points above.

Software and code

Policy information about availability of computer code

Data collection	No softwares were used for data collection.
Data analysis	BellCurve for Excel (Social Survey Research Information Co., Ltd.)

For manuscripts utilizing custom algorithms or software that are central to the research but not yet described in published literature, software must be made available to editors and reviewers. We strongly encourage code deposition in a community repository (e.g. GitHub). See the Nature Portfolio guidelines for submitting code & software for further information.

Data

Policy information about availability of data

All manuscripts must include a data availability statement. This statement should provide the following information, where applicable:

- Accession codes, unique identifiers, or web links for publicly available datasets
- A description of any restrictions on data availability
- For clinical datasets or third party data, please ensure that the statement adheres to our policy

The datasets generated during and/or analysed during the current study are available from the corresponding author on reasonable request.

Human research participants

Policy information about studies involving human research participants and Sex and Gender in Research.

Reporting on sex and gender	The research participants include both males and females.
Population characteristics	Healthy volunteers and individuals who had been diagnosed as kidney stone holders.
Recruitment	Co-authors (N.M., Y.S., Y.I. and K.T.) recruited the healthy volunteers and patients with a precise explanaton about the study and their agreements.
Ethics oversight	Informed consent in writing was obtained from donors of serum, and the study protocol conformed to the ethical guidelines of the 1975 Declaration of Helsinki as reflected in a priori approval by the Ethics Committees of the University of Tokyo for Medical Experiments (Permission Number: 2019358NI) and Tokyo Women's Medical University (Permission Number: 2020-0016).

Note that full information on the approval of the study protocol must also be provided in the manuscript.

Field-specific reporting

Please select the one below that is the best fit for your research. If you are not sure, read the appropriate sections before making your selection.

☒ Life sciences ☐ Behavioural & social sciences ☐ Ecological, evolutionary & environmental sciences

For a reference copy of the document with all sections, see nature.com/documents/nr-reporting-summary-flat.pdf

Life sciences study design

All studies must disclose on these points even when the disclosure is negative.

Sample size	The sample size was determined as sufficient for statistical analysis.
Data exclusions	No data were excluded from the analysis.
Replication	All attempts for replication were successful in this study.
Randomization	Randomization was not applied.
Blinding	Although the treatment of animals (e.g. adminstration of substances) was not carried out in a blind fashion, contributors who performed the animal treatments were not involved in the analysis of the samples isolated from the animals. The analysis was performed in a blind fashion.

Reporting for specific materials, systems and methods

We require information from authors about some types of materials, experimental systems and methods used in many studies. Here, indicate whether each material, system or method listed is relevant to your study. If you are not sure if a list item applies to your research, read the appropriate section before selecting a response.

Materials & experimental systems

n/a	Involved in the study	
☐	☒	Antibodies
☐	☒	Eukaryotic cell lines
☒	☐	Palaeontology and archaeology
☐	☒	Animals and other organisms
☒	☐	Clinical data
☒	☐	Dual use research of concern

Methods

n/a	Involved in the study	
☒	☐	ChIP-seq
☐	☒	Flow cytometry
☒	☐	MRI-based neuroimaging

Antibodies

Antibodies used	described in ONLINE METHODS section in the manuscript
Validation	Antibody validation was performed along the manufacture's protocols.

Eukaryotic cell lines

Policy information about cell lines and Sex and Gender in Research

Cell line source(s)	mProx cells, provided from the researcher who established the cells
Authentication	The cell line used for experiments were not authenticated.
Mycoplasma contamination	There was no Mycoplasma contamination.
Commonly misidentified lines (See ICLAC register)	No commonly misidentified cell lines were used in this study.

Animals and other research organisms

Policy information about studies involving animals; ARRIVE guidelines recommended for reporting animal research, and Sex and Gender in Research

Laboratory animals	Mouse, males , C57BL/6, and in-house generated AIM-deficient mice and KIM-1 deficient mice (on a C57BL/6 strain)
Wild animals	No wild animals were used in this study.
Reporting on sex	Male mice were used in this study.
Field-collected samples	No field-collected samples were used in this study.
Ethics oversight	The protocol was approved by the Committee on the Ethics of Animal Experiments of the University of Tokyo (Permit Number: P21-001).

Note that full information on the approval of the study protocol must also be provided in the manuscript.

Flow Cytometry

Plots

Confirm that:

☒ The axis labels state the marker and fluorochrome used (e.g. CD4-FITC).

☒ The axis scales are clearly visible. Include numbers along axes only for bottom left plot of group (a 'group' is an analysis of identical markers).

☒ All plots are contour plots with outliers or pseudocolor plots.

☒ A numerical value for number of cells or percentage (with statistics) is provided.

Methodology

Sample preparation	The mProx cells incubated with FITC-labeled CaOx crystals were washed and resuspended in PBS for analysis.
Instrument	FACSCelesta (BD)
Software	FACSDiva (BD) and FlowJo (BD)
Cell population abundance	100%
Gating strategy	gated in living cells based on the FSC/SSC profile

☒ Tick this box to confirm that a figure exemplifying the gating strategy is provided in the Supplementary Information.
